# Efficacy of a mobile technology-based intervention for increasing parents’ safety knowledge and actions: a randomized controlled trial

**DOI:** 10.1186/s40621-021-00350-w

**Published:** 2021-10-01

**Authors:** Lara B. McKenzie, Kristin J. Roberts, Rebecca J. McAdams, Mahmoud Abdel-Rasoul, Orie Kristel, Alison Szymanski, Sarah A. Keim, Wendy C. Shields

**Affiliations:** 1 Center for Injury Research and Policy, Abigail Wexner Research Institute at Nationwide Children’s Hospital, 700 Children’s Drive, Columbus, OH 43205 USA; 2 Department of Pediatrics, College of Medicine, The Ohio State University, 370 W. 9th Ave., Columbus, OH 43210 USA; 3 Division of Epidemiology, College of Public Health, The Ohio State University, 250 Cunz Hall, 1841 Neil Ave., Columbus, OH 43210 USA; 4 Center for Biostatistics, The Ohio State University, 320 Lincoln Tower, 1800 Cannon Dr., Columbus, OH 43210 USA; 5 Illuminology, 5258 Bethel Reed Park, Columbus, OH 43220 USA; 6 Bloomberg School of Public Health, Johns Hopkins Center for Injury Research and Policy, 624 N. Broadway, Baltimore, MD 21205 USA

**Keywords:** Multiple injury, Behavior change, Randomized trial, Child, Home, Safety

## Abstract

**Background:**

Leading causes of unintentional child injury such as poisoning and falls are preventable, and the majority occur in the home. Numerous home safety interventions have been developed and tested to increase safety behaviors; however, no smart phone-based applications (apps) have been developed and evaluated for this purpose. The objective of this study was to evaluate whether a mobile technology-based health behavior change intervention, the Make Safe Happen® app, was an effective tool to increase safety knowledge and safety actions/behaviors for the prevention of child unintentional injuries in and around the home.

**Methods:**

Data were collected in pretest and posttest online surveys from an existing nationwide population-based survey panel. Intervention subjects were randomized to organically (participant-driven) use the Make Safe Happen® app for 1 week, which provided home safety information and the ability to purchase safety products, while control participants were assigned to download and use an app about a topic other than home safety. The primary outcomes of safety knowledge and home safety actions were assessed by using linear mixed model regressions with intention-to-treat analyses.

**Results:**

A total of 5032 participants were randomized to either the intervention (*n* = 4182) or control (*n* = 850) group, with 2055 intervention participants downloading and entering their participant IDs into the Make Safe Happen® app. The online posttest survey was completed by 770 intervention and 283 control subjects. Mean knowledge parent safety score increased at a greater rate for intervention than control subjects (*p* < 0.0001), and at posttest was significantly higher for intervention than control subjects (*p* < 0.0001). The percentage of intervention subjects who reported doing all one-time and repeated safety actions significantly increased from pretest to posttest (*p* < 0.0001 and *p* = 0.0001, respectively), but there was no change among the control subjects (*p* = 0.1041 and *p* = 0.9755, respectively). At posttest, this percentage was larger for intervention than control subjects only for repeated safety actions (*p* = 0.0340).

**Conclusions:**

The mobile application significantly improved safety knowledge and safety actions for participants using the Make Safe Happen® app, although loss to follow-up was a limitation. The results of this study indicate the usefulness of widespread distribution and use of the Make Safe Happen® app.

*Trial registration number*NCT02751203; Registered April 26, 2016.

## Background

Child and adolescent unintentional injury, such as injuries caused by falls, fires, drownings, and poisonings, is a major public health problem in the United States (U.S.), consistently serving as the leading cause of mortality among children 1–19 years of age since 1981 [1, 2]. In 2018, approximately 6200 children died and 5.5 million children were treated in emergency departments for nonfatal injuries [3]. Most of these injuries were preventable and more than one half of unintentional injuries occur where children spend most of their time—in and around the home [4]. Parents and caregivers can mitigate these risks by removing hazards, installing safety devices, and practicing safe behaviors [5], such as storing medicines and household cleaners in locked cabinets [5, 6], properly installing and regularly using smoke alarms [5, 7], carbon monoxide detectors [8], stair gates [5, 9], and television and furniture anchors [5].

Despite the existence of known effective countermeasures to prevent home injury, the overall rates of safety device use and safety behaviors to prevent home injury such as locking up poisons and testing smoke alarms are unacceptably low. A 2015 survey of more than 1000 parents of children ≤ 12 years of age found that 30% kept medicines and cleaning products in an area accessible to their toddler, 14% had never checked their smoke alarm batteries, 13% had left their young child in a bathtub for at least 5 min without supervision, and 48% had not secured their televisions and furniture with anti-tip straps [10]. These low rates of safety behaviors may be explained by the lack of a centralized and easily accessible platform to access information on “child proofing” the home, or the perception that these actions are cumbersome, difficult, confusing, and time-consuming. Additionally, parents and caregivers often have difficulty identifying hazards in their home [11], finding credible information and recommendations, and obtaining the safety products best-suited to their home.

Previous interventions to reduce childhood injuries by increasing home safety practices most often included home safety education and/or the provision of safety equipment to parents or caregivers and were commonly conducted in-person via one-to-one sessions. These interventions have been shown to increase a range of safety practices that may reduce child injury rates [12, 13]; however, they are impractical for wide distribution because of the large amount of required resources [13]. With the near ubiquity of smartphones [14], smartphone applications (apps) are efficient, cost-effective, wide-reaching, readily-available, and are an effective means of delivering health information or behavior change recommendations [15]. It is possible that using an app to deliver home safety information and to track safety actions of parents and caregivers may be similarly effective. Furthermore, smartphone apps can offer targeted information on multiple safety topics via a single platform and can provide parents and caregivers with the ability to acquire child safety devices easily. To our knowledge, only one other home safety app (Safer Home) was created to decrease childhood injury in the home, but it has not been scientifically evaluated beyond basic usability and satisfaction measures [16].

The purpose of this study was to evaluate a mobile technology-based health behavior change intervention, the Make Safe Happen® app, to increase safety knowledge and safety actions/behaviors for the prevention of child unintentional injuries in and around the home. The hypothesis was that parents and caregivers of children ≤ 12 years who were randomly assigned to use the Make Safe Happen® app for 1 week would increase their safety knowledge and safety actions/behaviors compared to a control group who was assigned to use a non-injury app.

## Methods

Email invitations were sent to potential participants, who were members of a pre-existing, national online survey panel. Members of the panel were comprised of US adults enrolled by random-digit-dial telephone calls, US Postal Service mailings, and advertising on social networking websites. The panel utilized for this study was comprised of US adults who were parents of young children. The methods to recruit participants into the panel are heterogeneous ones employed by the consumer panel to continually update and recruit participants.

To be eligible, participants were required to: (1) have a smartphone with either an Android or iOS system and be willing to download and use an app; (2) be a parent or legal guardian of at least one child (referred to as the “index child”) aged 0–12 years; and (3) live with this index child “most of the time.” Participants who had previously downloaded or used the Make Safe Happen® app or the control app (Allrecipes Dinner Spinner app) were deemed ineligible (*n* = 2903) for the current study. All of the participants recruited from the panel for this study received an email invitation to complete an eligibility survey. A pilot study with *n* = 200 panel participants was completed prior to the full launch of the study to examine the distribution of participants’ responses and to address any technical difficulties with the survey instrument, programming, and data file. These data from *n* = 200 were included in the full analysis, including randomization, and the following numbers. A total of 21,478 potential participants opened the eligibility survey, 17,359 started the survey, 16,925 answered the first survey question, but did not complete the survey, and 6081 completed the screener and were considered eligible to participate. A total of 434 people were assigned to a condition, but never started the survey. The 6081 participants who were deemed eligible for study inclusion were invited to complete the pretest online survey, of which 5032 completed the pretest survey. The study was approved by the Institutional Review Board at Nationwide Children’s Hospital (Assurance #: FWA00002860, Expiration Date: 06/15/21, Registration #: IRB00000568, Institution #: IORG0000326). Consent to participate was obtained for each study participant prior to enrollment via online consent form.

All participants who started the pretest survey (*n* = 17,359) were randomized by using a computer-generated number system in a 5:1 ratio to either the intervention group (to use the Make Safe Happen® app, version 1.0.3, *n* = 4182), or the control group (to use the Allrecipes Dinner Spinner app, *n* = 850). The 5:1 ratio was selected to maximize the number of intervention group participants and minimize control condition participants who were asked to use a “sham” app for which download status was unable to be confirmed. Randomization was blocked in groups of five based on the index child’s age in age subgroups: 0–11 months, 12–23 months, 2–4 years, 5–9 years, and 10–12 years, but no additional factors. After completion of their pretest survey, participants were emailed and asked to download the Make Safe Happen® app or Allrecipes Dinner Spinner app from iTunes or GooglePlay app stores, at no cost, onto their smartphone, to use for about one week, and then to organically (i.e., exposure to the app information was participant-driven) complete a posttest survey.

Respondents indicated their consent to participate on an online consent form. Participants who completed all parts of the study, in both conditions, were compensated as a thank you for their time by a point rewards system (equivalent to $8–$10). These data were collected in the United States from July 2016 through September 2016.

### Index child

The “index child” was identified for each participant via a least filled quota protocol, meaning a participant was assigned to a child age group depending on the needs, or quotas, for each age group for the entire sample and treatment group. When a new participant with more than one child between 0 and 12 years of age completed a pretest survey, that participant was assigned to a quota age group based on whichever had the smallest number of completed participant surveys at that time. The selection of an “index child” was only relevant for assigning participants to child age groups for recruitment to prevent unequal child age groups in the treatment groups. Birth order was not used as a determinant for assigning participants to child age groups.

### Pretest and posttest assessment

Online pretest and posttest surveys measured safety knowledge and safety behaviors and were administered before and after a 1-week app use period to all participants. The pretest and posttest surveys were completed in approximately 10–15 min and were very similar, differing only in the posttest where the intervention group was asked to answer 8 additional questions about their app experience.

#### **Make Safe Happen**® **app: intervention condition**

The Make Safe Happen® app was developed to help parents and caregivers of children 0–12 years of age learn how to make their homes safer for their children. General features of the Make Safe Happen® app included the ability to receive safety information by child’s age group (0–11 months, 12–23 months, 2–4 years, 5–9 years, and 10–12 years), with room-by-room safety checklists and links to purchase common home safety products on Amazon.com. App features also included the ability for users to create shopping lists for safety products, set reminders for testing and replacing batteries in safety devices, and to add the National Capital Poison Center’s Poison Control Helpline emergency phone number for poison control to their contacts [17]. Once downloaded, app users had the option to receive notifications through the app and were not required to purchase safety products. For more information on the Make Safe Happen® app and the study methods, readers are referred to a full description of the study protocol [18].

#### Participant identification and tracking actions in the Make Safe Happen® app using Google Analytics

Intervention group participants were asked to enter an assigned participant identification (ID) number, which was provided to them via email upon completion of the pretest survey, into the Make Safe Happen® app. Participants in the intervention group received email reminders about downloading the Make Safe Happen® app at 24- and 72 h after pretest completion. The ID number established a link between the pretest and posttest surveys of each intervention group study participant and their unique app actions by using Google Analytics (GA). GA is a free Web-based analytic platform that allowed intervention group study participants who entered their ID into the Make Safe Happen® app to have their app actions, such as setting reminders to test safety devices, adding safety products to a shopping list, and checking off safety actions, tracked. For example, if an intervention group participant used the Make Safe Happen® app to check off safety actions they completed, the actions were reported in GA, and then could be compared to the participant’s pretest and posttest survey response relating to those corresponding items.

#### Allrecipies Dinner Spinner app: control condition

Participants randomly assigned to the control condition (*n* = 850) were asked to download and use the Allrecipes Dinner Spinner app, (version 6.1) which was described as “the most popular food—focused social app” that helps “cooks discover and share the joy of home cooking” (App Store, Food and Drink, All Recipies, Inc. Allrecipes.com). Download status and use of the control group app by the control group participants was not confirmed. The control group participants were invited to download the intervention app after completing their posttest survey. Unlike the intervention subjects, control subjects who downloaded the Allrecipes Dinner Spinner app did not have their app actions followed in GA.

### Measures and statistical analysis

Primary outcomes for the present evaluation included: (1) safety knowledge; and (2) safety actions. Participants self-reported these at baseline (pretest) and at followup (posttest). A combination of yes–no, multiple choice and Likert scale response option items were used. A sample of questions from the pretest and posttest surveys were vetted and revised if needed for coherent meaning and level of difficulty via an hour-long cognitive interview with *n* = 20 parents prior to the launch of the study.

#### Safety knowledge

The mean total safety knowledge score was calculated for pretest and posttest intervention group, and pretest and posttest control group participants. To calculate the mean total safety knowledge score, safety knowledge was measured by responses to 17 questions created specifically for this study and were based on safety messages and content that was delivered in the Make Safe Happen® app. One point was given for each correct answer and correct responses were summed to determine a total knowledge score for each participant.

#### Safety actions (one-time safety actions vs. repeated safety actions)

Safety actions (either one-time or repeated) were measured by responses to 29 questions. Participants were asked about 14 safety behaviors that are recommended to do repeatedly, e.g., turning pot handles away from the stove when cooking, and 15 safety behaviors that are typically completed once, e.g., buying and installing a carbon monoxide detector. For example participants were presented the question stem, “I repeatedly take the following safety actions in my home…” and were then presented with several statements such as, “Turn pot handles to the back of the stove when I cook.” Response choices included the following options: always, sometimes, never, and not applicable. Based on these response choices, two dichotomous variables were created, one for one-time safety actions, and the other for repeated safety actions. These variables indicated if (1) participant “always” or “sometimes” took the action for all responses, and (2) participants “never” took the action for all responses; “not applicable” was set to missing.

#### Demographic information

Basic demographic information including participant’s gender, age, highest level of education completed, employment status, number of parents per household, home ownership, time living at current address, household income, ability to make ends meet, and age and gender of children < 18 years living in the home were collected on the pretest survey. The age(s) of all the participants’ child(ren) (not only the index children) were categorized for analysis into three groups: (1) younger children only (i.e., all children for each participant were < 5 years of age); (2) older children only (i.e., all children for each participant were ≥ 5 years of age ); and (3) younger and older children only (i.e., each participant had children in both the < 5 years and ≥ 5 years age groups.

### Statistical analysis

Data analysis consisted primarily of two main activities: (1) the generation of descriptive information summarizing the sample and its characteristics; and (2) the analyses related to the study aims and hypotheses. Descriptive data were considered first, both in the full sample and within the intervention and control groups. Differences across intervention and control groups were evaluated as well as adherence to the protocol, defined as completing both surveys and downloading and using the app (intervention group: validated by participant ID via GA). Using an intention-to-treat analysis, the primary hypothesis was tested by using linear mixed model regression on the following outcome measures: safety knowledge (mean safety knowledge) and safety actions (one-time and repeated). Primary outcomes for the safety behaviors and device use were assessed while stratifying by the index child’s age group. Exposure analyses were conducted to examine the relationship between outcomes and exposure to the intervention. Intervention group participants’ data for those who downloaded the Make Safe Happen® app were linked with their GA data. Statistical significance was assessed using *α* = 0.05.

### Power calculation

Our team originally identified two scenarios of acceptable power sample sizes that differed by different acceptable effect estimates. First, we determined a sample of 800 participants at posttest would achieve 80% power to detect a mean of paired differences of 1 with an estimated standard deviation of differences of 10 at 0.05 significance level. Second, we found that a sample of 1200 subjects at posttest would achieve 80% power to detect a mean of paired differences of 0.8 with similar estimated standard deviation of differences and significance level as in the first scenario. Our final posttest sample size (*n* = 1053) allowed us to achieve acceptable power with an effect estimate of 1. The power analysis assumed knowledge score as the primary outcome.

## Results

**Fig. 1:**
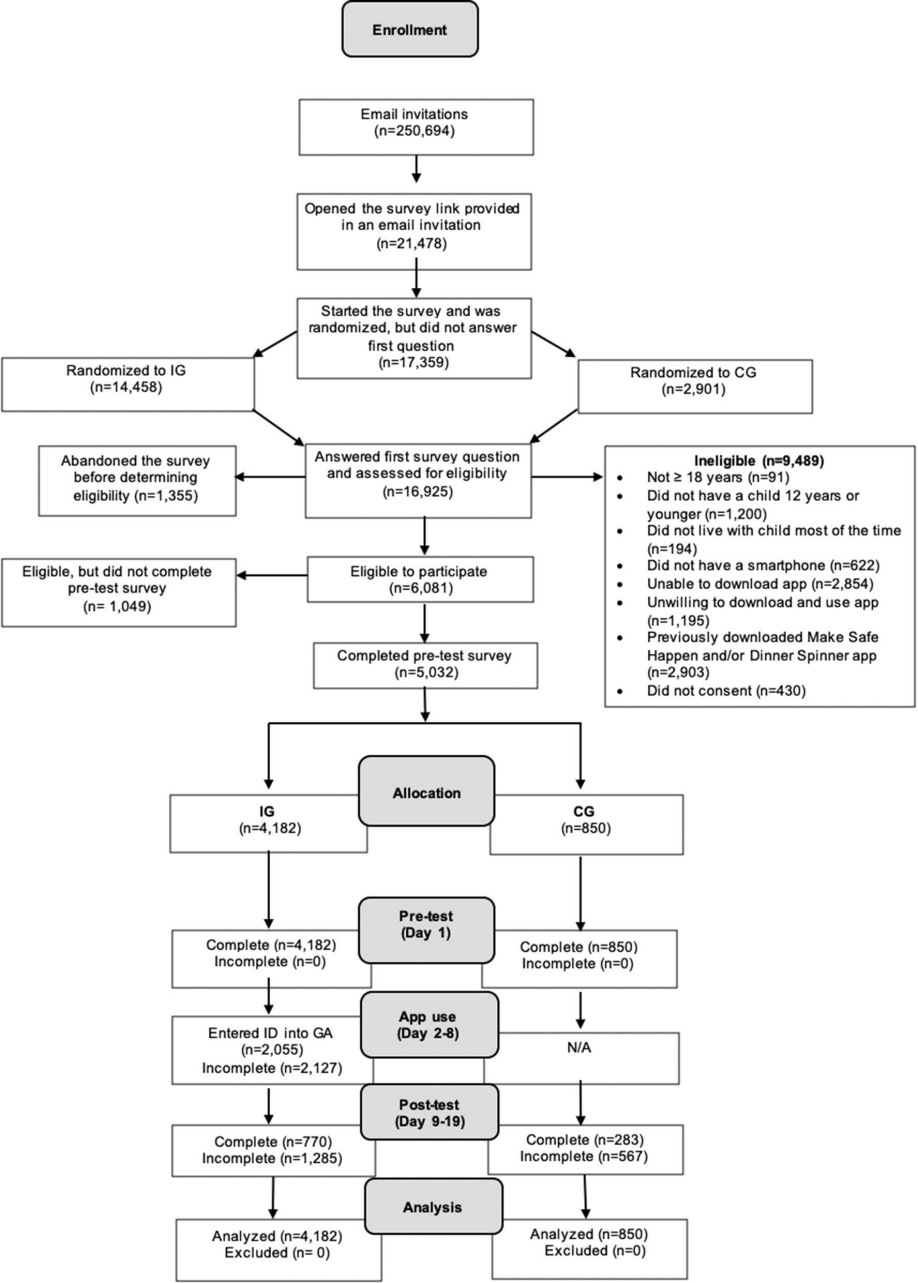
CONSORT flowchart of study enrollment

Figure 1 illustrates the CONSORT flowchart of participation and attrition for the current study, including the number of completed surveys and app downloads confirmed (intervention group only) for the study sample. Overall, a total of 5032 parent participants completed the online pretest survey. A total of 4182 were randomized to the intervention and 850 were randomized to the control. Of those assigned to the intervention, a total of 2055 were confirmed to have downloaded the Make Safe Happen® app and entered a participant ID code. Of those who downloaded the Make Safe Happen® app and entered a participant ID code, 770 completed the online posttest survey. Of those randomized to the control, a total of 283 completed the online posttest survey.

Table 1 lists demographic characteristics of the sample, both overall and by randomly-assigned study condition. Most participants were White, non-Hispanic (71.7%), female (67.9%), with at least a Bachelor’s degree (57.0%), owned their home (67.4%) for 5 years or more (42.2%), and lived in a two-parent household (76.8%). The mean age of participants was 34.7 years (SD = 7.5).

**Table 1:** Study sample demographic characteristics

	Pretest			Posttest		
	Total sample^a^	IG^a,b^	CG^a,c^	Total sample^a^	IG^a,b^	CG^a,c^
	*n* = 5032	*n* = 4182 *n* (%)	*n* = 850	*n* = 1053	*n* = 770 *n* (%)	*n* = 283
Age (years), mean (SD)	34.7 (7.5)	34.6 (7.4)	34.8 (7.7)	35.6 (7.5)	35.7 (7.7)	35.3 (6.7)
Number of children < 18 in household, mean (SD)	2.0 (1.0)	2.0 (1.0)	2.1 (1.0)	2.1 (1.1)	2.1 (1.1)	2.1 (1.1)
Gender						
Male	1614 (32.1)	1344 (32.1)	270 (31.8)	269 (25.6)	158 (20.5)	111 (39.2)
Female	3418 (67.9)	2838 (67.9)	580 (68.3)	784 (74.5)	612 (79.5)	172 (60.8)
Index child age						
0–11 months	775 (15.4)	632 (15.1)	143 (16.8)	147 (14.0	116 (15.1)	31 (11.0)
12–23 months	790 (15.7)	669 (16.0)	121 (14.2)	175 (16.6)	139 (18.1)	36 (12.7)
2–4 years	1156 (23.0)	954 (22.8)	202 (23.8)	266 (25.3)	196 (25.5)	70 (24.7)
5–9 years	1153 (22.9)	967 (23.1)	186 (21.9)	220 (20.9)	151 (19.6)	69 (24.4)
10–12 years	1158 (23.0)	960 (23.0)	198 (23.3)	245 (23.3)	168 (21.8)	77 (27.2)
Child age						
Young only (< 5 years)	1254 (24.9)	1033 (24.7)	221 (26.0)	288 (27.4)	230 (29.9)	58 (20.5)
Mix young and old	1521 (30.2)	1266 (30.3)	255 (30.0)	306 (29.1)	224 (29.1)	82 (29.0)
Old only ( > = 5 years)	2257 (44.9)	1883 (45.0)	374 (44.0)	459 (43.6)	316 (41.0)	143 (50.5)
Race						
White	4147 (82.4)	3460 (82.7)	687 (80.8)	881 (83.7)	644 (83.6)	237 (83.8)
Black	505 (10.0)	412 (9.9)	93 (10.9)	88 (8.4)	61 (7.9)	27 (9.5)
Asian/Pacific Islander	213 (4.2)	163 (3.9)	50 (5.9)	53 (5.0)	38 (4.9)	15 (5.3)
Native American or Alaskan Native	44 (0.9)	42 (1.0)	2 (0.2)	11 (1.0)	9 (1.2)	2 (0.7)
Other	123 (2.4)	105 (2.5)	18 (2.1)	20 (1.9)	18 (2.3)	2 90.7)
Ethnicity						
Hispanic/Latino	695 (13.8)	573 (13.7)	122 (14.4)	128 (12.2)	83 (10.8)	45 (15.9)
Non-Hispanic/Latino	4337 (86.2)	3609 (86.3)	728 (85.7)	925 (87.8)	687 (89.2)	238 (84.1)
Education^d^						
≤High school/GED	775 (15.4)	643 (15.4)	132 (15.5)	124 (11.8)	100 (13.0)	24 (8.5)
Some college	1387 (27.6)	1155 (27.6)	232 (27.3)	314 (29.9)	253 (32.9)	61 (21.6)
≥Bachelor’s degree	2868 (57.0)	2382 (57.0)	486 (57.2)	614 (58.4)	416 (54.1)	198 (70.0)
Employment						
Full time	2953 (58.7)	2465 (58.9)	488 (57.4)	572 (54.3)	396 (51.4)	176 (62.2)
Part time	572 (11.4)	474 (11.3)	98 (11.5)	122 (11.6)	84 (10.9)	38 (13.4)
Stay at home parent	1182 (23.5)	969 (23.2)	213 (25.1)	307 (29.2)	250 (32.5)	57 (20.1)
Not employed	114 (2.3)	99 (2.4)	15 (1.8)	20 (1.9)	14 (1.8)	6 (2.1)
Other	211 (4.2)	175 (4.2)	36 (4.3)	32 (3.0)	26 (3.4)	6 (2.1)
Income^e,f^						
< $20,000	341 (6.9)	279 (6.8)	62 (7.5)	52 (5.1)	43 (5.7)	9 (3.3)
$20,000–$39,999	863 (17.5)	747 (18.1)	116 (14.1)	173 (16.8)	143 (19.0)	30 (10.8)
$40,000–$59,999	854 (17.3)	699 (17.0)	155 (18.8)	200 (19.4)	157 (20.9)	43 (15.5)
$60,000–$79,999	1048 (21.2)	897 (21.8)	151 (18.4)	208 (20.2)	152 (20.2)	56 (20.2)
≥ $80,000	1839 (37.2)	1500 (36.4)	339 (41.2)	397 (38.5)	258 (34.3)	139 (50.2)
Livability (ability to make ends meet)^g^						
With great difficult	393 (7.9)	343 (8.3)	50 (6.0)	49 (4.7)	37 (4.9)	12 (4.3)
With difficulty	491 (9.9)	410 (9.9)	81 (9.6)	96 (9.2)	78 (10.3)	18 (6.4)
Just get by	1801 (36.2)	1502 (36.3)	299 (35.6)	429 (41.2)	328 (43.2)	101 (35.9)
Easily	1601 (32.2)	1320 (31.9)	281 (33.5)	336 (32.3)	235 (30.9)	101 (35.9)
Very easily	692 (13.9)	563 (13.6)	129 (15.4)	131 (12.6)	82 (10.8)	49 (17.4)
Home ownership						
Own	3391 (67.4)	2806 (67.1)	585 (68.8)	729 (69.2)	507 (65.8)	222 (78.5)
Rent	1567 (31.1)	1316 (31.5)	251 (29.5)	310 (29.4)	250 (32.5)	60 (21.2)
Other	74 (1.5)	60 (1.4)	14 (1.7)	14 (1.3)	13 (1.7)	1 (0.4)

^a^Some categories do not total 100% because of rounding.

^b^IG = Intervention group

^c^CG = Control group

^d^There are missing education levels for pretest: total sample (*n* = 2), IG (*n* = 2), and for posttest: total sample (*n* = 1), IG (*n* = 1)

^e^There are missing income values for pretest: total sample (*n* = 87), IG (*n* = 60), CG (*n* = 27), and for posttest: total sample (*n* = 23), IG (*n* = 17), CG (*n* = 6)

^f^At pre-test, *p* < 0.01; At post-test, *p* < 0.0001

^g^There are missing livability values for pretest: total sample (*n* = 54), IG (*n* = 44), CG (*n* = 10), and for posttest: total sample (*n* = 12), IG (*n* = 10), CG (*n* = 2)

### Effect of the intervention on safety knowledge

Mean parent safety knowledge score for between intervention and control at pretest and posttest (Table 2) were compared. At pretest, there was no statistical difference in the mean knowledge score between intervention (8.45) and control subjects (8.51; *p* = 0.6225). Mean knowledge score significantly increased between the pretest and posttest for both intervention (8.45–10.32; *p* < 0.0001) and control participants (8.51–8.87; *p* = 0.0064). This increase occurred at a greater rate for those in the intervention group compared to those in the control group (*p* < 0.0001). At posttest, mean knowledge score for intervention subjects (10.32) was significantly larger than that of control subjects (8.87; *p* < 0.0001).

**Table 2:** Mean knowledge score, percent all one-time safety actions complete, percent all repeated safety actions complete by study group and pretest and posttest

	Adjusted for child age group			No adjustment		
	Pretest	Posttest	*p* value	Pretest	Posttest	*p* value
Mean knowledge score						
IG	n/a	n/a	n/a	8.45	10.32	< 0.0001
CG	n/a	n/a	n/a	8.51	8.87	0.0064
All one-time safety actions						
IG	52.9%	60.7%	< 0.0001	51.8%	60.2%	< 0.0001
CG	51.1%	55.8%	0.1041	50.1%	54.3%	0.1598
All repeated safety actions						
IG	71.1%	77.3%	0.0001	71.7%	77.7%	0.0002
CG	70.9%	71.0%	0.9755	71.6%	72.0%	0.8826

### Child age group and knowledge score

**Fig. 2:**
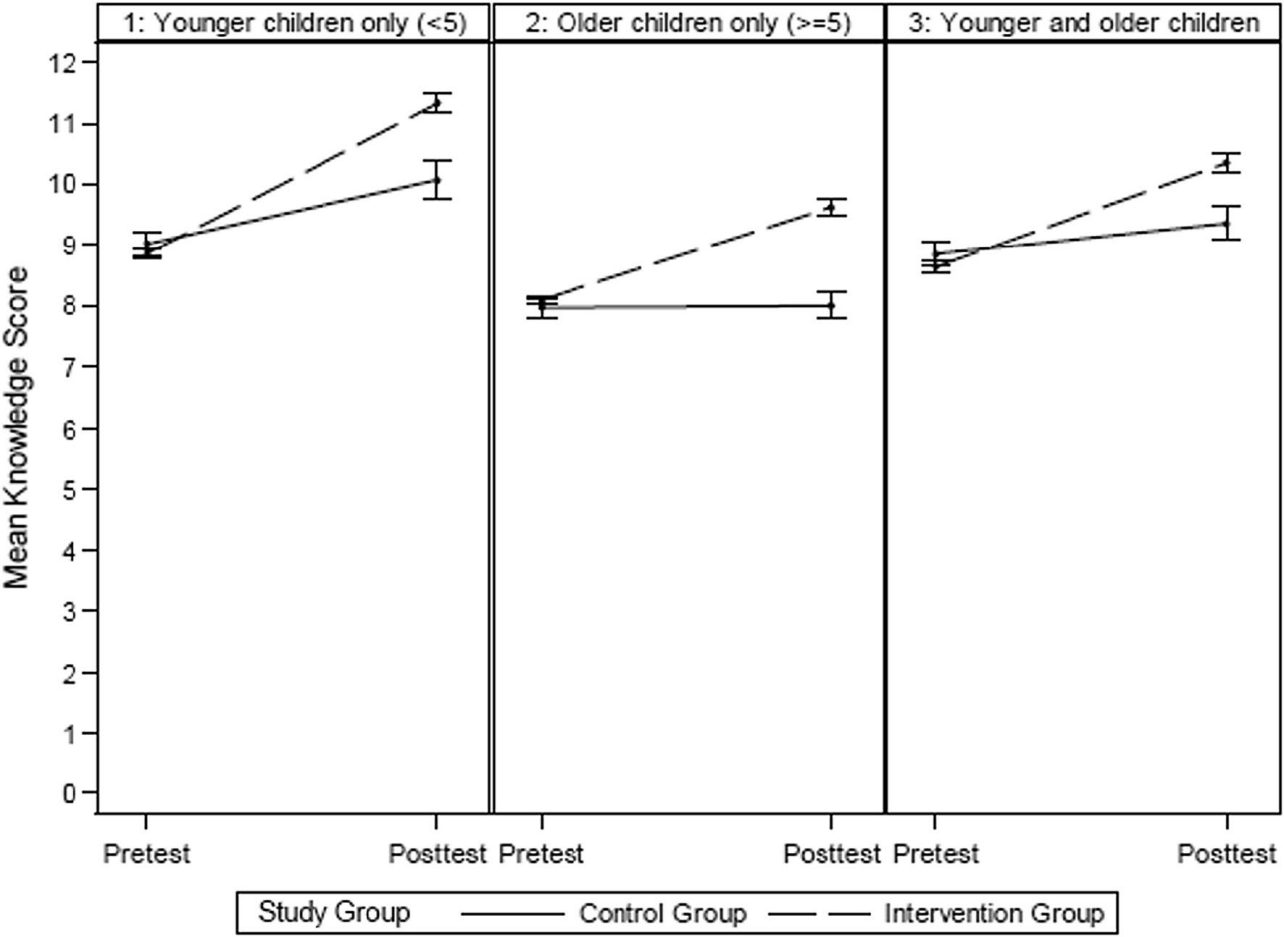
Mean knowledge score by child age, study group, and pretest and posttest

Figure 2 shows the mean knowledge score at pretest and posttest for the intervention and control subjects across all child age groups. After adjusting for child age groups, the mean knowledge score increased from pretest to posttest for intervention and control participants, but the increase was 1.37 units higher on average (95% CI 1.06, 1.68; *p* < 0.0001) in the intervention than the control. Among parents who only have young children (< 5 years), the increase in mean knowledge score from pretest to posttest was 1.41 units greater on average (95% CI 0.78, 2.03; *p* value < 0.0001) for those in the intervention than those in the control. For parents who only have older children (≥ 5 years), the increase in posttest knowledge scores relative to pretest knowledge score was 1.50 units greater on average (95% CI 1.07, 1.93; *p* value < 0.0001) for the intervention group than the increase for the control group. Among parents who have old and young children, the increase in mean knowledge score between pretest and posttest was 1.20 units greater on average (95% CI 0.65, 1.75; *p* value < 0.0001) for intervention versus control subjects.

The increase in mean knowledge score for intervention participants was greatest among parents who only have young children. Among participants, the increase in mean knowledge score between pretest and posttest was significantly higher for parents who only have young children than the increase among parents who only have older children (0.92 units on average, 95% CI 0.57, 1.30, *p* < 0.0001) and parents who have both young and older children (0.76 units on average, 95% CI 0.36, 1.16, *p* = 0.0002). The increase in mean posttest knowledge scores and mean pretest knowledge scores did not differ significantly between parents with older children and parents with both young and older children (*p* = 0.3555).

### Effect of the intervention on one-time safety actions

The percentage of intervention subjects who reported taking all safety actions significantly increased from 52.9% at pretest to 60.7% at posttest (*p* < 0.0001), but there was no change in percentage among control participants (*p* = 0.1041; Table 2). The posttest percentage of intervention participants who did all one-time safety actions was not significantly larger than that of control participants (55.8%; *p* = 0.1584). Among participants who did all one-time safety actions at pretest and had a posttest, a higher percentage of participants move in the “right” direction (from not doing to doing all safety actions) in the intervention group (44.0%) compared to the control group (29.1%). Among participants who did all one-time safety actions at pretest and had a posttest, a higher percentage of participants moved in the “wrong” direction (from doing all to not doing all safety actions) in the control (17.6%) compared to the intervention group (11.4%).

After child age was adjusted for in the analysis, we found that the increase in the proportion of participants who performed one-time safety actions from pretest to post was significant for intervention subjects (*p* < 0.0001), but not significant for control subjects (*p* = 0.1596). There was no statistical difference in posttest proportion between the intervention and control participants (*p* = 0.0846), and there was no difference in the rate of change from pretest to posttest between the intervention and control groups (*p* = 0.2119). The percentage of one-time safety actions was largest for parents with only older children (58.9%; 95% CI 56.3%, 61.5%), followed by parents with young and older children (55.8%; 95% CI 52.9%, 58.8%), and parents with only younger children (47.6%; 95% CI 44.4%, 50.7%). The difference in percentages between parents with only younger children compared to parents with only older children and with both young and older children were significant (*p* < 0.0001 for both), while the difference in percentages between parents with only older children compared to young and older children was marginally significantly (*p* = 0.0546).

### Effect of the intervention on repeated safety actions

Overall, 71.1% of all respondents did all repeated safety behaviors at pretest, and 75.6% of participants who completed both pre- and posttests did all repeated safety behaviors at posttest, indicating a larger proportion of posttest participants did all repeated safety behaviors at posttest compared to pretest. The percentage of participants who reported doing all repeated safety actions significantly increased from 71.1% at pretest to 77.3% at posttest for the intervention group (*p* = 0.0001), while there was no significant change in percentage (70.9% at pretest to 71.0% at posttest) among control subjects who did all repeated safety actions (*p* = 0.9755; Table 2). Furthermore, the posttest percentage of intervention participants who did all repeated safety actions was larger than that of control participants (*p* = 0.0340). Among participants who did not do at least one repeated safety action at pretest, a higher percentage of participants moved in the “right” direction (from not doing all repeated safety action at pretest to doing all repeated safety actions at posttest) in the intervention group (52.9%) compared to the control group (41.5%). Among participants who did all repeated safety actions at pretest, a higher percentage of control participants moved in the ‘wrong’ direction (from doing all repeated safety actions at pretest to not doing at least one repeated safety actions at pretest; 9.5%) compared to intervention participants (9.1%). Of these participants who moved in the “wrong” direction, a higher percentage intended to do the behavior in the future among the control (88.9%) compared to the intervention group (48.9%).

After adjusting for child age group, the change from pretest to posttest among the intervention group is significant (*p* = 0.0002), but is not significant among the control group (*p* = 0.8826). At posttest, the difference between the intervention and control groups is marginally significant (*p* = 0.0501). The rate of change from pretest to posttest was not significantly different, but marginally different, between intervention and control subjects (*p* = 0.0632). We also found that child age group is a significant predictor of repeatedly taking the safety action (*p* < 0.0001). The proportion of participants repeatedly doing the action was largest for parents who have both young and older children (76.0%; 95% CI 73.4%, 78.5%), followed by parents who have only young children (74.1%; 95% CI 71.3%, 76.6%), and parents who have only older children (69.7%; 95% CI 67.2%, 72.1%). There was no statistical difference in proportions between parents of young and older children and parents of only young children (*p* = 0.2126), but the differences between parents who have only younger versus only older children (*p* 0.0041), and between parents who have only older versus young and older aged children (*p* < 0.0001) were significant.

### Google Analytics

The *n* = 2055 intervention participants who entered their ID in the Make Safe Happen® app performed 77,506 app actions (Table 3). Nearly 69,000 (*n* = 68,797) safety items were checked off in the app. There were 933 actions where “shop for item” was selected in the app, 630 and 255 occasions where a safety item was added to the shopping list or on Amazon, respectively. Participants performed over 400 (*n* = 419) actions to set reminders to test safety devices, and shared a safety tip directly from the app on social media on 55 occasions. If participants checked off all safety items within a room in the app, then that room was considered complete; because each participant had only one opportunity to complete each room, the number of room completes directly reflects the number of participants who completed that action. Approximately 43% (43.1%; *n* = 886) of intervention group participants completed at least one room in the app. The laundry room was most often completed (34.1%; *n* = 700), followed by kitchen (29.8%; *n* = 613) and bedroom (29.4%; *n* = 605).

**Table 3:** Make Safe Happen® app behavior for IG study participants

Randomized into IG	4182
MSH app participant IDs entered	2055
Participants with age selected	1948
iOS	781
Android	1280
Selected child age in app	
0–11 months	358
12–23 months	395
2–4 years	743
5–9 years	1016
10–12 years	648
Rooms completed (any room)	886
Bathroom completed	356
Bedroom completed	605
Kitchen completed	613
Living room completed	109
Laundry room completed	700
Basement completed	515
Stairs and hallways completed	289
Total room completes	3187
Mark all done	3819
Social share	55
Added poison help number to contacts	200
Set reminder, total	419
For testing smoke alarms	76
For testing carbon monoxide alarms	32
For replacing smoke alarm batteries	46
For replacing carbon monoxide alarm batteries	30
Check off	68,797
Shop for	933
Shop for shopping list	630
Shop for Amazon	255
Total actions/clicks	77,506

## Discussion

The current study evaluated the Make Safe Happen® app’s impact on safety knowledge and safety actions among parents and caregivers of children ≤ 12 years of age. Mobile technology and the proliferation of smartphones has opened up a new avenue for reaching crucial target audiences. The Make Safe Happen® app was created to help parents and caregivers make their homes safe for their children, by helping them to identify home safety hazards, complete home safety actions, and acquire and install home safety devices. The intention of the app was  to increase safety knowledge and the number of safety actions, ultimately reducing the number of deaths and injuries sustained by children in and around the home. Results from the current study indicate that parents and caregivers can learn about home safety and take safety actions after using the Make Safe Happen® app which is easy to administer and disseminate. At the time of this study, few mobile apps exist aimed to prevention unintential injuries in and around the home and none have been similarly or as rigorously evaluated.

As is true with other preventive health behaviors, successful adoption of the behavior, such as safety actions, is comprised of a complex set of steps and actions. Knowledge about a topic is an essential first step in the cascade of events that lead to behavior adoption. From this study, the Make Safe Happen® app proves to be an effective tool to increase safety knowledge among parents and caregivers. In particular, this study’s findings indicate that knowledge change in parents of only younger children (< 5 years of age) benefitted the most from app use compared to parents of only older and both younger and older children, exhibiting the greatest increase in their mean knowledge score from pretest to posttest. This may be because parents of only younger children, more so than parents who have older children, are still forming their beliefs, gaining knowledge, and may have more room to grow in their awareness of their home safety practices as well as their child’s abilities and inabilities.

Achievement of “safety actions” was measured in a couple of ways in the current study. First by self-report as part of the pretest and posttest surveys and second by recorded app actions (actions taken within the app and recorded as GA data). For the pretest and posttest data, both repeated and one-time safety actions increased significantly for parents in the intervention group, but there was a discrepancy in increase of safety actions among parents of different aged children. For an increase in one-time safety actions (i.e., buying and installing carbon monoxide detectors), the MSH app proved most beneficial for parents of only older children; however for an increase in repeated safety actions (i.e., turning pot handles away from the stove), parents who have both younger and older children benefited the most from the app. This could be because there are more safety actions that need to be done for children of younger ages than children of older ages.

For this study GA proved to be a useful tool to track data points within the app, allowing us to not be solely reliant on self-report surveys that may be limited due to respondant bias. GA is also less cumbersome, time-consuming, and resource-heavy than home visits to observe safety actions, which have been typically used in home safety assessments for child injury prevention research [19, 20]. GA can be an efficient resource for researchers to track data and actions for participants using apps.

While unintentional child injuries and deaths remain high and parent safety behaviors relatively low [1–3, 10], an easy-to-implement and effective intervention is needed. The increase in safety knowledge and actions signify that the Make Safe Happen® app is an effective intervention, even after only 1 week’s use. Parents who used the Make Safe Happen® app achieved significant positive changes in their knowledge about home safety and completed more safety actions to prevent home-related child injuries than those who did not use the Make Safe Happen® app. The benefits of the app to parents and caregivers varies by child age. With the ability to target messages in the app to parents and caregivers of specific child ages, varying benefits can be achieved. The advantage of this model of intervention and message delivery is that it does not have to be a one size fits all intervention.

### Limitations

This study had some limitations. There was a large amount of loss to follow-up in both the intervention (81.6%) and control (66.7%) groups, which may lead to bias. Although this is a large percentage, it is similar to the low to follow-up present in other studies using similar methodology [21, 22]. We compared the characteristics of those who did complete and those who did not complete the study. There were differences by age, gender, education, employment, income and livability. These differences may indicate the presence of loss to follow-up bias, where our final sample differs from the enrolled sample. We also compared the characteristics between those who completed the study (completed posttest) and those who did not complete the posttest, stratified by intervention group and control group. The intervention group differed by age, gender, index child age, child age group, ethnicity, education, employment, income, and livability. The control group differed by gender, index child age, child age group, education, employment, income, and home ownership. These differences indicate the possibility of loss to follow-up bias, which could lead to biases in the results.

Further, there may be some learning bias present from taking a similar survey twice relatively close in time, which may explain why there was no difference in proportion of participants completing one-time safety actions at posttest. The intervention and control group were “attention matched” that is, both intervention group and control group were asked to download and use an app for a period of 7–10 days. No restrictions nor encouragement were included for either group on app use. The only difference in surveys for both groups were the addition of items about MSH app use for the  intervention group. Despite these efforts the authors recognize that biases may arise.

Time between pretest and posttest was relatively short, approximately 1 week, which participants reported in past research was too little time to adopt new safety actions. Perhaps giving participants more time to use the app would enable them to complete more safety actions, however with added app use time there was a risk of additional loss to follow-up. Furthermore, the control group participants did not have to input an ID into the control app, which could have presented a bias of more engaged participants in the intervention group. Safety actions were self-reported and were not confirmed by the study via observation. Although there is a tendency to overreport correct behavior when using self-report methodologies, actions checked off in the app may translate directly that the action was fully achieved. In our previous work involving focus groups [23], parents reported that they only checked off the safety action in the app after the action was complete. New apps or programs should consider the best way to engage parents and caregivers to help them achieve safety actions. Future work to evaluate the Make Safe Happen® app on parent and caregiver safety actions might also consider other aspects and barriers of completing home safety actions such as difficulties with installation of products (the need for tools, skill, and time), acquisition and purchase (cost) of safety products, adoption of behaviors over time, and challenges of sustaining these safety actions and behaviors over time. At posttest, participants in this study indicated that they did not take safety actions in the past week because they already follow the home safety recommendations, they intend to make changes in the near future, they did not feel the information was relevant to them, they could not afford home safety products, and they did not have time to make the changes. Future studies should further investigate how to help parents overcome these barriers and accomplish actions which could help caregivers prevent child injuries in their homes. Future studies should also examine best and most successful practices to disseminate a mobile home app broadly, so that parents are aware of the resource.

Despite these limitations, this study had several strengths, including the use of a randomized controlled design which involves random assignment to intervention or control conditions, multiple outcome measures, baseline assessment, and a large sample size to evaluate the hypotheses.

## Conclusions

The results of this study offer initial promise for smartphone apps to increase caregiver knowledge and home safety actions which could lead to the prevention of child injuries in and around the home. Despite the proliferation of health behavior apps, this is one of the first studies to investigate a home safety app using a randomized trial. This evidence sufficiently demonstrates the utility of this mobile app for unintentional child home-related injury prevention.

## Data Availability

The dataset generated and analyzed during the current study are not publically available but are available from the corresponding author on reasonable request.

## Abbreviations

app: Application
CI: Confidence interval
U.S.: United States
CONSORT: Consolidated standards of reporting trials
iOS: iPhone operating system
ID: Identification
